# An evaluation of STROBE reporting compliance in cross-sectional studies published in the *Journal of Korean Biological Nursing Science* (2011–2024): a methodological review

**DOI:** 10.7586/jkbns.25.089

**Published:** 2026-02-27

**Authors:** Mi-Kyoung Cho, Mi Young Kim

**Affiliations:** 1 Department of Nursing Science, College of Nursing, Chungbuk National University, Cheongju, Korea; 2 College of Nursing, Hanyang University, Seoul, Korea

**Keywords:** Cross-sectional studies, Nursing research, Guideline adherence, Quality control

## Abstract

**Purpose:**

This study aimed to evaluate item-level reporting compliance with the Strengthening the Reporting of Observational Studies in Epidemiology (STROBE) checklist among cross-sectional studies published in the *Journal of Korean Biological Nursing Science* (JKBNS) between 2011 and 2024, and to identify both strengths and gaps in reporting practices.

**Methods:**

A descriptive methodological review was conducted. Using the official JKBNS online archive, 192 cross-sectional survey studies published between 2011 and 2024 were identified and included in the analysis. Reporting compliance was evaluated using the STROBE checklist, and item-level mapping was performed between STROBE items and the Joanna Briggs Institute and National Institutes of Health (NIH) critical appraisal critical appraisal tools.

**Results:**

The mean overall STROBE compliance rate was 74.0%. Reporting of study objectives, outcome data, and interpretation was generally adequate across the included studies. However, substantial deficiencies were observed in the Methods domain. In particular, sample size justification, reporting of bias, handling of missing data, description of sampling strategies, sensitivity analyses, and inclusion of participant flow diagrams were rarely reported.

**Conclusion:**

The reporting quality of cross-sectional studies published in JKBNS was moderate, with notable weaknesses in methodological transparency. These deficiencies may limit the interpretability and applicability of research findings in nursing practice and policy contexts. Systematic and explicit application of the STROBE reporting guidelines, supported by guideline-based education and strengthened editorial policies, is essential for improving reporting transparency and methodological rigor in nursing research.

## INTRODUCTION

### 1. Background

Nursing research plays a critical role in providing the scientific foundation for nursing practice and contributing to the development of healthcare policies [1]. The value of scientific research depends on the credibility and reproducibility of its findings, which begin with transparent and complete reporting of research methods and results [2]. In particular, observational studies are subject to less control than experimental studies and are therefore more vulnerable to bias, making clear and detailed reporting of study design, conduct, and analysis especially important [3]. However, many studies fail to report essential methodological information adequately or completely [4,5], which may limit the interpretation and application of research findings.

To address these challenges, international reporting guidelines have been developed to improve the quality of research reporting. The EQUATOR (Enhancing the QUAlity and Transparency Of health Research) Network provides a comprehensive collection of reporting guidelines tailored to various study designs [6]. The adoption of reporting guidelines enhances transparency, completeness, and reproducibility of research, improves the quality of systematic reviews and meta-analyses, and ultimately strengthens the foundation of evidence-based practice [7,8]. Indeed, several studies have demonstrated a positive association between adherence to reporting guidelines and the overall quality of published articles [9,10]. Therefore, evaluating the extent to which reporting guidelines are followed is essential.

One of the most frequently employed research designs in nursing is the descriptive survey study, among which cross-sectional studies are particularly useful for examining participants’ characteristics, health status, attitudes, and behaviors at a specific point in time and for exploring relationships among variables [11,12]. Cross-sectional studies can be conducted within a relatively short time frame and at lower cost, and they are effective in identifying risk factors for diseases or health-related problems [13]. In addition, cross-sectional studies play an important role in nursing research by facilitating the exploration of emerging phenomena, generating hypotheses, and providing foundational data for subsequent longitudinal or interventional studies [14]. Despite these advantages, cross-sectional studies have inherent methodological limitations, including difficulty in inferring causal relationships, inability to establish temporal sequences, and increased susceptibility to bias [15]. Therefore, it is particularly important to clearly recognize these limitations and to report the design, conduct, analysis, and interpretation of cross-sectional studies accurately and transparently.

The international reporting guideline for cross-sectional studies is the Strengthening the Reporting of Observational Studies in Epidemiology (STROBE) statement. Developed in 2007, the STROBE guidelines have been established as an international standard for improving the reporting quality of observational studies [6]. The STROBE statement provides a structured checklist designed to enhance the quality of reporting in cross-sectional studies and to help researchers systematically address key information that is frequently omitted [16]. However, studies examining adherence to the STROBE guidelines indicate that compliance remains suboptimal across many academic journals. Systematic reviews of international medical and nursing journals have reported that only approximately 50%~70% of STROBE checklist items are adequately reported, with particularly poor reporting observed for items related to clear description of study design, sample size justification, methods for controlling bias, and handling of missing data [17,18]. Therefore, there is a clear need to assess journal-specific adherence to international reporting guidelines for cross-sectional studies.

The quality of an academic journal is largely determined by the methodological rigor and transparency of reporting in the articles it publishes, which in turn directly influence the journal’s impact, citation rates, and international recognition [19]. Despite the availability of established reporting guidelines, systematic evaluations of how faithfully published articles adhere to these guidelines remain limited. In Korea, nursing journals have experienced substantial quantitative growth over the past several decades, accompanied by a steady increase in the number of journals indexed by the National Research Foundation of Korea [20]. Since its establishment in 1999, the *Journal of Korean Biological Nursing Science* (JKBNS) has served as a leading journal in basic nursing research, contributing to the strengthening of the scientific foundation of nursing through the publication of interdisciplinary studies integrating basic medical sciences—such as physiology, pathology, pharmacology, microbiology, and anatomy—with nursing science [21].

However, improving journal quality requires more than an increase in publication volume; it necessitates a systematic examination of research designs and levels of evidence. In particular, there has been a lack of comprehensive analysis regarding the extent to which articles published in the JKBNS adhere to international reporting guidelines appropriate to their respective study designs. Therefore, this study aims to evaluate the quality of published articles based on established reporting guidelines and to propose practical strategies for enhancing the overall quality of research reporting in the journal.

Meanwhile, the methodological quality of cross-sectional studies is commonly assessed using appraisal tools developed by the Joanna Briggs Institute (JBI) [22] and the National Institutes of Health (NIH) [23]. However, these tools primarily focus on evaluating the rigor of study design and conduct, and thus differ in purpose and scope from reporting guidelines such as STROBE. Accordingly, mapping STROBE checklist items to the JBI and NIH appraisal criteria at the item level can provide important insights into the conceptual overlap and distinctions between reporting guidelines and methodological quality assessment tools, thereby clarifying the respective roles and limitations of each instrument.

Accordingly, this study aimed to systematically assess the reporting quality of cross-sectional studies published in the JKBNS from 2011 to 2024 using the STROBE reporting guideline. Furthermore, item-level mapping between the STROBE checklist and the JBI and NIH appraisal tools was performed. The findings of this study are intended to inform practical strategies to enhance the reporting quality of articles published in the journal.

### 2. Study aim

This study aimed to evaluate item-level reporting compliance with the STROBE checklist in cross-sectional studies published in the JKBNS between 2011 and 2024, and to identify strengths and gaps in reporting practices. In addition, this study mapped STROBE checklist items at the item level to the JBI and NIH cross-sectional study appraisal tools. This study seeks to provide empirical evidence on the current level of reporting transparency in cross-sectional studies published in JKBNS and to offer practical insights for improving reporting standards.

## METHODS

### 1. Study design

This study was conducted as a descriptive methodological review to evaluate the reporting quality of cross-sectional survey studies published in JKBNS from 2011 to 2024, based on the STROBE checklist.

### 2. Data source and search strategy

The literature search for this study was conducted from November 10 to November 15, 2025. The data source was the official online archive of JKBNS (https://jkbns.org/articles/archive.php). For this study, all articles published in JKBNS between 2011 and 2024 were retrieved, and a comprehensive dataset was constructed using Microsoft Excel (Office 365; Microsoft Corporation, Redmond, WA, USA). The dataset included bibliographic information such as publication year, authors, article title, DOI, and study design. During the initial screening phase, titles and abstracts were reviewed using the keywords “cross-sectional,” “survey,” and “descriptive study.” Out of a total of 479 published articles, 192 were identified as cross-sectional survey studies and were considered potentially eligible for inclusion.

### 3. Eligibility criteria

**Figure 1.**
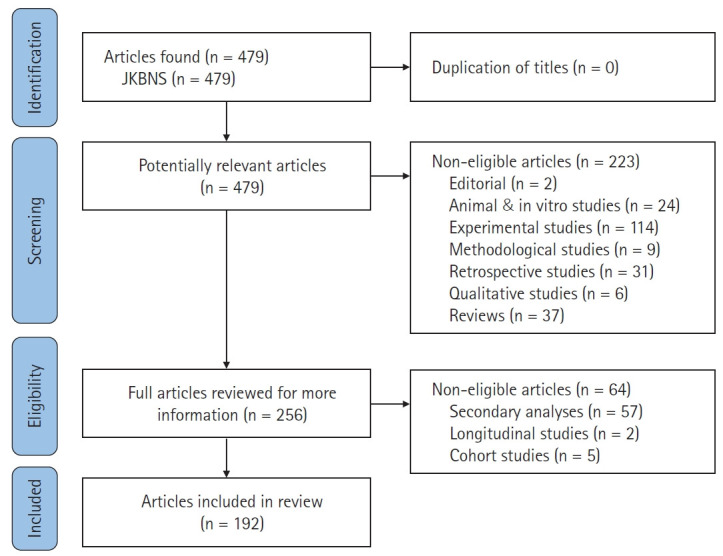
Flowchart of study selection. JKBNS = *Journal of Korean Biological Nursing Science*.

Studies were included in the analysis if they met the following criteria: (1) published in JKBNS between 2011 and 2024; and (2) explicitly identified as cross-sectional survey studies through the use of terms such as “cross-sectional,” “survey,” or “descriptive study” in the title, abstract, or methods section. Studies were excluded if they met any of the following criteria: (1) review articles, including literature reviews, integrative reviews, comprehensive reviews, or brief reviews; (2) experimental studies, such as randomized controlled trials or quasi-experimental designs; (3) studies involving animal subjects; (4) studies based on secondary data analysis; and (5) cohort studies. The study selection process is illustrated in Figure 1.

### 4. Instruments

#### 1) STROBE checklist

Although observational studies are widely conducted in health care research, concerns have repeatedly been raised that published reports often provide insufficient information to appraise the strengths and limitations of the findings and to judge their generalizability [6]. The STROBE development process was initiated through a consensus-based approach at a workshop held in September 2004, which involved methodologists, researchers, and journal editors; the checklist was subsequently finalized through iterative revisions informed by empirical evidence and methodological considerations [24]. STROBE was developed to cover three major observational study designs—cohort, case–control, and cross-sectional studies—and its final output is a 22-item checklist [6]. The STROBE checklist is organized to align with the conventional structure of research articles across six domains: title/abstract (item 1), introduction (items 2,3), methods (items 4~12), results (items 13~17), discussion (items 18~21), and other information (item 22). Of the 22 items, 18 apply to all study designs, whereas four items (6,12,14, and 15) are design-specific, with reporting requirements that vary by cohort, case–control, and cross-sectional studies [6]. Several items are further subdivided into sub-items (e.g., a–e), and when these sub-items are treated as individual assessment units for the cross-sectional version, the checklist comprises a total of 32 evaluable sub-items [25]. Importantly, STROBE is intended to facilitate sufficiently complete and transparent reporting of conducted studies rather than to prescribe how studies should be designed or carried out [25]. In this study, adherence to each STROBE item was assessed as “yes” when fully reported, “no” when not reported, and “NA” when not applicable; “yes” and “no” were scored as 1 and 0 points, respectively, and NA items were excluded from scoring. The reporting adherence score (or proportion) was calculated for each article using only applicable items as the denominator [26]. In the present review, only the cross-sectional study version of STROBE was applied.

#### 2) JBI Critical Appraisal Checklist for Analytical Cross-Sectional Studies

JBI provides standardized critical appraisal checklists tailored to specific study designs to systematically assess the methodological quality of included studies, particularly in the context of evidence synthesis, where the risk of bias is a central concern. Among these, the JBI Critical Appraisal Checklist for Analytical Cross-Sectional Studies is designed to evaluate the potential for bias in analytical cross-sectional studies that examine etiology, risk factors, or associations between exposures and outcomes. This checklist was developed by JBI in collaboration with methodological experts, reviewed by its Scientific Committee, and refined through extensive peer consultation. The tool consists of eight items assessing key methodological domains, including clarity of inclusion criteria (item 1), adequacy of descriptions of study participants and setting (item 2), validity and reliability of exposure measurement (item 3), use of objective and standardized criteria for condition measurement (item 4), identification and management of confounding factors (items 5,6), validity and reliability of outcome measurement (item 7), and appropriateness of statistical analysis (item 8). These items are structured to evaluate common sources of bias in analytical cross-sectional designs, such as selection bias, information bias, and confounding, as well as the overall rigor of data analysis. Each item is rated using one of four response options: Yes, No, Unclear, or Not applicable (NA) [22].

#### 3) NIH Quality Assessment Tool for Observational Cohort and Cross-Sectional Studies

The National Heart, Lung, and Blood Institute (NHLBI), a component of the NIH, has developed and disseminated study design–specific Study Quality Assessment Tools to support evidence synthesis and guideline development by systematically evaluating internal validity and the risk of bias. These tools are intended to identify potential methodological and implementation-related sources of bias (e.g., selection, performance, attrition, measurement/detection bias, and confounding) and to inform an overall judgement of study quality based on the extent to which such limitations may influence interpretation of the findings [23]. In the present study, methodological quality was appraised using the Quality Assessment Tool for Observational Cohort and Cross-Sectional Studies, which comprises 14 items covering: clarity of the research question/objective (item 1); study population and recruitment (definition of the population, participation rate, recruitment from the same population/time period, and consistency of eligibility criteria; items 2~4); justification of sample size or power considerations (item 5); temporality and adequacy of the observation time frame (items 6,7); assessment of exposure levels (e.g., dose–response; item 8); validity and reliability of exposure measurement (item 9); repeated assessment of exposure (item 10); validity and reliability of outcome measurement (item 11); blinding of outcome assessors (item 12); loss to follow-up when applicable (item 13); and measurement and statistical control of key confounders (item 14) [27]. Each item is rated as Yes, No, or Other, where Other includes cannot determine (CD), not reported (NR), and NA; item-level judgements are then synthesized into an overall rating of Good/Fair/Poor, with Good indicating the lowest risk of bias and highest confidence in validity, Fair indicating some susceptibility to bias that is unlikely to invalidate the results, and Poor indicating substantial limitations with a meaningful risk of bias [7]. Given that exposures and outcomes are frequently assessed at the same time point in cross-sectional studies, the NHLBI guidance recommends rating the temporality question (item 6) and the time frame question (item 7) as “No” for cross-sectional analyses in which temporal precedence cannot be established [23].

### 5. Data collection

Data for this study were obtained from the official online archive of JKBNS and systematically organized in a data extraction spreadsheet developed in Microsoft Excel (Office 365; Microsoft Corporation). The spreadsheet captured bibliographic information for all articles published between 2011 and 2024, including author(s), publication year, article title, DOI, and study design. Reasons for exclusion were recorded at each stage of the screening process to ensure traceability, with separate documentation for the first-stage screening (title/abstract review) and the second-stage screening (full-text review). To enhance the accuracy of study design classification, two reviewers independently screened all records using predefined eligibility criteria. During the first screening stage, potentially eligible articles were identified based on titles and abstracts, and full texts were subsequently reviewed in the second stage to determine final inclusion. Discrepancies primarily arose when (1) the cross-sectional survey design was not explicitly stated, (2) it was unclear whether the study was based on primary survey data versus secondary data analysis, or (3) the design could be interpreted as involving follow-up and thus resembling a cohort approach. Such disagreements were resolved through discussion and consensus, informed by methodological descriptions within each article (e.g., study design, participant recruitment, data collection procedures, and analytic plans). For studies included in the final sample, study characteristics were extracted using a standardized template, including Study ID, author(s), publication year, title, study design, sample size, participant characteristics (e.g., sex, age, group), setting, number of independent variables, and statistical methods. One reviewer conducted the initial extraction, and a second reviewer cross-checked the extracted information to correct errors and confirm the final dataset. STROBE-based reporting assessments were conducted by two reviewers. Prior to the main evaluation, the reviewers performed a pilot assessment of 10 articles to calibrate interpretation and establish item-level decision rules. All eligible articles were then assessed independently, and any inconsistencies in item ratings were resolved through 2~3 rounds of discussion until consensus was reached. Throughout the selection and assessment procedures, decisions were documented step-by-step to support methodological transparency and reproducibility. In addition, to ensure conceptual coherence between STROBE reporting items and the methodological quality appraisal tools (JBI and NIH), the two reviewers independently conducted a structured item-mapping procedure and reconciled disagreements through discussion. Specifically, each STROBE item (including cross-sectional sub-items) was examined and compared against corresponding items in the JBI Critical Appraisal Checklist for Analytical Cross Sectional Studies and the NIH Quality Assessment Tool for Observational Cohort and Cross-Sectional Studies to determine conceptual equivalence or correspondence. The final mapping was confirmed through mutual review and consensus.

### 6. Data analysis

Data analysis was conducted to systematically summarize the general characteristics of the included observational studies and to assess the completeness of reporting in accordance with the STROBE checklist. In addition, the results of the reporting assessment were examined in relation to the conceptual domains of established observational study quality appraisal tools, namely the JBI Cross-sectional checklist and the NIH Cross-sectional tool, in order to explore areas of conceptual alignment and distinction between reporting guidelines and methodological quality assessment frameworks.

Study characteristics were analyzed using descriptive statistics, including frequencies, percentages, means, and standard deviations. For the assessment of STROBE reporting items, checklist items that were NA were excluded from score calculation. Items that were fully reported were coded as 1, whereas items that were partially reported or not reported were coded as 0. For STROBE items comprising two or more sub-items, an item-level score was calculated as the mean of the corresponding sub-item scores. Item-level STROBE adherence scores were then calculated for each study by summing the item-level scores across all applicable items, and overall reporting performance was summarized using the mean and standard deviation across studies. In addition, the proportion of STROBE items adequately reported was calculated as the percentage of fully reported items relative to the total number of applicable items after excluding NA items. Finally, individual STROBE items were conceptually mapped to the corresponding quality assessment domains of the JBI Cross-sectional checklist and the NIH Cross-sectional tool to compare domains in which reporting completeness and methodological quality overlapped or diverged. Both the STROBE item-level assessment and the conceptual mapping process were independently performed by two reviewers, and inter-rater agreement was subsequently calculated to evaluate the consistency of the assessments.

### 7. Ethical considerations

This study was conducted as a secondary review using data obtained exclusively from previously published articles and did not involve direct interaction with human participants or the collection of new, identifiable individual-level data. Accordingly, Institutional Review Board (IRB) approval was not required, in line with ethical standards governing reviews of publicly available research materials. All information extracted from the included studies was appropriately referenced to ensure transparency, proper attribution of original sources, and adherence to principles of academic integrity. To reduce the risk of selection or extraction bias, the processes of study selection and data extraction were independently carried out by two reviewers, with discrepancies resolved through discussion.

## RESULTS

### 1. Overview of cross-sectional studies published in JKBNS from 2011 to 2024

A total of 192 cross-sectional studies published in the JKBNS between 2011 and 2024 were included in the analysis (Table 1). By publication period, 82 articles (42.7%) appeared between 2011 and 2015, followed by 67 (34.9%) between 2016 and 2020, and 43 (22.4%) between 2021 and 2024. In terms of study design, the majority were descriptive studies (166, 86.5%), whereas 24 (12.5%) adopted correlational designs. Only one article (0.5%) explicitly reported the use of a reporting guideline. IRB approval was documented in 153 articles (79.7%), and 64 (33.3%) reported receiving research funding. Regarding participant characteristics, 146 articles (76.0%) included both women and men, and 154 (80.2%) focused on adults aged 18 years or older. With respect to participant type, the general population was most frequently examined (71, 37.0%), followed by healthcare professionals and trainees (64, 33.3%) and patients (46, 24.0%); 11 studies (5.7%) did not involve human participants. The mean sample size was 188.0 ± 124.8 participants (range: 6~956), with 121 (63.1%) recruiting fewer than 200 participants. Nearly half were conducted in hospital settings (95, 49.5%), followed by school settings (29.7%) and community or institutional settings (15.6%). The mean number of settings was 3.22 ± 8.32 (range: 1~80), and 107 (55.8%) were carried out in a single setting. The mean number of independent variables examined was 2.36 ± 1.33, with 119 (62.0%) investigating fewer than three independent variables. More than half included a single dependent variable (112, 58.3%). In terms of outcome domains, health-related behaviors (49, 25.5%) were most frequently assessed, followed by physiological and clinical outcomes (30, 15.6%), psychological and psychosocial outcomes (27, 14.1%), and quality of life and well-being (14, 7.3%). Regarding statistical approaches, simple and multiple regression analyses were most commonly applied (74, 38.5%), followed by correlation analyses (41, 21.4%), group difference analyses (39, 20.3%), and logistic regression analyses (25, 13.0%).

### 2. Assessment of reporting quality based on the STROBE checklist

Table 2 summarizes STROBE item-level reporting compliance among 192 cross-sectional studies published in JKBNS between 2011 and 2024. In the title and abstract domain, the abstract (item 1b) was reported in all studies (100.0%), whereas the title (item 1a) was reported in only 6 studies (3.1%). Both background/rationale (item 2) and objectives (item 3) were consistently reported across all studies. Substantial variability was observed in the Methods domain. While study design (item 4; 99.0%), setting (item 5; 88.5%), and data sources/measurement (item 8; 100.0%) were frequently reported, eligibility criteria (item 6; 60.4%), sample size justification (item 10; 50.0%), and particularly bias (item 9; 9.9%) were infrequently reported. Among statistical method sub-items, subgroup or interaction analyses (item 12b) were reported in 89.6% of studies, whereas handling of missing data, sampling strategy, and sensitivity analyses (items 12c~12e) were not reported in any study (0.0%). Confounding control (item 12a) was reported in 30.2% of studies. In the Results domain, outcome data (item 15) were fully reported in all studies (100.0%), and unadjusted effect estimates (item 16a) and category boundaries (item 16b) were reported in 96.4% and 80.2%, respectively. Reporting of participant flow was incomplete, with flow diagrams (item 13c) reported in only one study (0.5%). In the Discussion domain, key results (item 18) and interpretation (item 20) were consistently reported (100.0%), whereas limitations (item 19; 47.9%) and generalizability (item 21; 51.0%) were reported in approximately half of the studies. Funding information (item 22) was reported in 37.5% of studies. The mean study-level STROBE score was 20.56 ± 2.27 (range: 13~25), and inter-rater agreement was almost perfect (Cohen’s κ = 0.99).

Table 3 summarizes reporting compliance with the 22 STROBE checklist items. The mean study-level STROBE reporting compliance rate was 74.0% ± 7.8% (range: 52.3%~90.3%). Overall, core items within the title/abstract, introduction, results, and discussion domains demonstrated high levels of reporting. In particular, background/rationale, objectives, outcome data, key results, and interpretation items were reported in all studies, corresponding to a compliance rate of 100.0%. In contrast, several items within the Methods domain showed limited reporting. Eligibility criteria were reported in 60.4% of studies, and sample size justification in 50.0%, whereas reporting of bias was notably low at 9.9%. With respect to statistical methods, items related to handling of missing data, sampling strategy, and sensitivity analyses were not reported in any study (0.0%). In addition, reporting of participant flow showed a compliance rate of 54.2%, and reporting of funding information was limited to 37.5% of studies.

### 3. Mapping STROBE checklist items to the JBI and NIH cross-sectional checklists

Table 4 presents the results of the item-level mapping between the STROBE checklist and two methodological quality appraisal tools, the JBI and the NIH cross-sectional checklist. STROBE items in the Methods domain were mapped to multiple items in both appraisal tools, particularly those related to study setting, participant selection, variable measurement, bias, and statistical methods. Within the Introduction domain, item 3 (objectives) was mapped to item 1 of the NIH tool. In the Methods domain, item 5 (setting) was mapped to item 2 of the JBI checklist and to multiple items in the NIH tool, and item 6 (eligibility criteria) was mapped to items 1 and 2 of the JBI checklist and to items 2 and 4 of the NIH tool. With respect to measurement-related items, item 7 (clearly define all outcomes) and item 8 (data sources/measurement) were mapped to corresponding measurement-related items in both the JBI and NIH tools. Item 9 (bias) showed one-to-many mappings with both appraisal tools. In the JBI checklist, this item was mapped to items addressing participant selection, confounding, outcome measurement, and statistical analysis, and in the NIH tool, it was mapped to items addressing participation rate, outcome measurement, blinding, and confounding adjustment. Item 10 (study size) was mapped to item 5 of the NIH tool, and item 11 (quantitative variables) was mapped to item 4 of the JBI checklist and item 8 of the NIH tool. Item 12 (statistical methods) was mapped to multiple analysis-related items in both appraisal tools. Within the Results domain, item 13 (participants flow diagram) was mapped to items related to participation rate and loss to follow-up in the NIH tool, and items 14~16 (descriptive data, outcome data, and main results) were mapped to result- and analysis-related items in both appraisal tools. In contrast, STROBE items in the Discussion domain (items 18~21) did not have direct counterparts in either the JBI or NIH tools. Item 22 (funding) was mapped to the funding-related item in the NIH tool.

Inter-rater agreement for the item-level mapping was high, with Cohen’s kappa values of κ = 0.85 for the JBI mapping and κ = 0.90 for the NIH mapping, indicating substantial to excellent agreement between the two independent reviewers.

## DISCUSSION

This study aimed to evaluate the level of compliance with each item of the STROBE checklist for cross-sectional studies published in JKBNS from 2011 to 2024 and to identify the strengths and weaknesses in reporting. Furthermore, by mapping STROBE checklist items to the JBI and NIH cross-sectional study assessment tools at the item level, it sought to provide empirical evidence on the current state of reporting transparency and derive implications for improving reporting standards.

Based on the findings of this study, the following points can be discussed. Only 0.5% of the papers explicitly reported the use of the STROBE reporting guidelines. This suggests that despite the guidelines being recommended as the international standard for reporting cross-sectional studies, they are not sufficiently recognized or institutionalized in the actual nursing research field. Furthermore, the finding that only 3.1% of papers in this study explicitly stated the research design in the title aligns with previous research indicating low compliance rates for design specification in titles [27]. When the research design is not specified in the title, it can hinder the visibility and usability of the study. Therefore, it is necessary to ensure that the design is clearly stated in the title.

The results in the Methods and Results sections were relatively low in this study. This aligns with previous research indicating that the average compliance scores were lowest in the Results section (36%) and Methods section (49%) [28]. A cross-sectional study has recommended improving transparency, particularly in the Methods and Results sections [29]. Furthermore, the average STROBE reporting compliance rate for research units in this study ranged from 52.3% to 90.3%, showing significant variation. This is similar to findings from a study on STROBE compliance in observational studies published in medical journals, where compliance rates varied from 24% to 68% [28].

Meanwhile, the average STROBE reporting compliance rate among the research units in this study was 74.0%. This was relatively high compared to a previous study analyzing 147 observational studies related to COVID-19 treatment published in Medline in 2020, which reported an average STROBE compliance rate per paper of 45.6% [30]. Furthermore, it was higher than the STROBE compliance rate reported in a previous study evaluating 223 randomly selected articles from three EAACI (European Academy of Allergy and Clinical Immunology) journals, where the overall complete STROBE reporting rate for cross-sectional studies was 45.6% [27].

At the item level, the present study found particularly low reporting rates for bias, with only 9.9% of studies addressing this item. Moreover, none of the included studies reported on the handling of missing data, sampling strategies, or sensitivity analyses. Control of confounding factors was reported in only 30.2% of the studies. These findings are consistent with previous studies that have highlighted insufficient reporting of sensitivity analyses, bias control, and missing data management in observational research [28]. Similar results were also reported in an evaluation of observational studies among Master of Public Health (MPH) theses indexed in Wanfang between January 2014 and May 2019, which identified low compliance rates for methodological and results-related items, including handling of missing data (6.7%), sensitivity analyses (3.6%), participant flow diagrams (15.2%), and reporting of absolute risk (0%) [29].

When reporting on the handling of missing data and sensitivity analyses, as required by the STROBE guidelines, is omitted, the assessment of internal validity and transparency of result interpretation is substantially limited. In particular, without clear information on the patterns and management of missing data, the potential for selection bias cannot be adequately ruled out. Likewise, the absence of sensitivity analyses prevents evaluation of the robustness of the analytical results. These limitations pose a risk of overgeneralization when findings from nursing research are applied to clinical practice or policy decision-making. Therefore, future cross-sectional studies in nursing should adhere more systematically to the STROBE reporting guidelines to enhance transparency and methodological rigor.

The results of this study indicate that reporting on subject flow was insufficient, with only 0.5% of studies presenting a flow diagram. This finding aligns with previous research highlighting inadequate reporting of flow diagrams [28,31]. When the participant recruitment and exclusion processes are not clearly presented, it becomes difficult to assess the potential for selection bias, and limitations arise in the representativeness and reproducibility of the final analysis sample. Particularly in nursing research, where participant heterogeneity is high and research findings are frequently utilized for clinical and policy decision-making, the importance of reporting participant flow charts as recommended by the STROBE guidelines is further emphasized.

The papers evaluated in this study were found to lack sufficient coverage of certain items recommended by the STROBE guidelines. This indicates a deficiency in key elements necessary for readers to assess the validity and applicability of the research [28]. As recommended in the 2020 report that the overall reporting quality of observational studies was weak and required improvement efforts from editors, reviewers, and authors [30], this suggests that educational interventions targeting specific items are necessary.

This study empirically demonstrated the conceptual overlap and distinctions between reporting guidelines and methodological quality assessment tools by comparing and mapping STROBE checklist items with the JBI and NIH cross-sectional study quality assessment tools at the item level. Specifically, the STROBE Method domain corresponded to multiple core items in both quality assessment tools, while items in the Discussion domain showed no direct mapping to either quality assessment tool. The finding that Methods domain items in STROBE were mapped to multiple items in the JBI and NIH tools in one-to-one or one-to-many relationships suggests that key elements of study design and conduct are closely associated with reporting completeness. This implies that studies adhering more rigorously to the STROBE guidelines are more likely to achieve higher ratings in methodological quality assessments.

In contrast, items within the Discussion domain of the STROBE checklist were not directly mapped to either the JBI or NIH quality appraisal tools. This finding suggests that existing methodological quality assessment instruments do not systematically evaluate the transparency of reporting related to result interpretation, acknowledgment of study limitations, and considerations of generalizability. This gap has important implications within the context of nursing research. Nursing research extends beyond the identification of causal relationships and is often conducted to inform clinical practice, support person-centered care, and guide policy and educational decision-making [32,33]. Given these characteristics, transparent reporting of result interpretation, explicit recognition of study limitations, and clear discussion of the generalizability of findings are critical elements that determine not only the scientific merit of research but also its clinical and policy relevance.

Accordingly, nursing journals should actively promote adherence not only to methodological quality appraisal tools but also to the Discussion domain of the STROBE guidelines in order to improve the reporting quality of cross-sectional studies. In addition, while the STROBE guidelines provide a framework for assessing reporting transparency, adherence to these guidelines should not be interpreted as being synonymous with the internal validity or methodological rigor of the original studies.

The present study is subject to certain considerations. The analysis was confined to articles published in a single journal, and adherence to the STROBE reporting guideline should be interpreted carefully, as reporting compliance does not necessarily reflect the internal validity or methodological rigor of the original studies. In addition, changes in STROBE compliance over time were described descriptively, without formal statistical testing of temporal trends. Future research may benefit from the application of longitudinal or time-series approaches to more rigorously examine changes in reporting practices over time.

## CONCLUSION

This study evaluated the reporting fidelity of cross-sectional studies published in JKBNS from 2011 to 2024 based on the STROBE checklist and mapped it to the JBI and NIH cross-sectional study assessment tools at the item level. The results showed that while core elements such as study purpose, main results, and interpretation were mostly reported faithfully, reporting imbalances were prominent in the methodology domain. Specifically, reporting on participant selection criteria, sample size justification, consideration of bias, missing data handling, sensitivity analysis, and participant flow diagrams was very limited. This may compromise the transparency of the research process and the validity of result interpretation. The finding that many STROBE items are linked to several methodological items in the JBI and NIH assessment tools suggests that faithful adherence to reporting guidelines is closely related to the methodological quality assessment of research.

In summary, to improve the quality of cross-sectional studies published in JKBNS, a more systematic and explicit application of the STROBE reporting guidelines is necessary. To achieve this, submission guidelines should explicitly reflect compliance with the reporting guidelines, and an education and monitoring system based on these guidelines should be implemented for authors and reviewers. The accumulation of these efforts may substantially contribute to improving the transparency of reporting in cross-sectional studies in nursing.

## Figures and Tables

**Table 1.** Characteristics of Cross-sectional Studies Published in JKBNS (2011~2024) (N = 192)

Characteristics		n (%)	M ± SD (range)
Publication year	2011~2015	82 (42.7)	
	2016~2020	67 (34.9)	
	2021~2024	43 (22.4)	
Type of studies	Descriptive studies	166 (86.5)	
	Correlational studies	24 (12.5)	
	SEM studies	2 (1.0)	
The reporting guideline stated	Yes	1 (0.5)	
	No	191 (99.5)	
IRB	Yes	153 (79.7)	
	No	39 (20.3)	
Fund	Yes	64 (33.3)	
	No	128 (66.7)	
Sex of the participants	Women	22 (11.5)	
	Men	5 (2.6)	
	Both	146 (76.0)	
	Not reported or not human	19 (9.9)	
Age of the participants (years)	< 18	8 (4.2)	
	≥ 18	154 (80.2)	
	≥ 65	17 (8.8)	
	Not reported or not human	13 (6.8)	
Samples	Healthcare professionals and trainees	64 (33.3)	
	Patients	46 (24.0)	
	General population	71 (37.0)	
	Not human	11 (5.7)	
Number of samples	< 100	28 (14.7)	188.03 ± 124.83 (6~956)
	100 ~ 199	93 (48.4)	
	200 ~ 299	43 (22.4)	
	≥ 300	24 (12.5)	
	Not reported	2 (1.0)	
Setting	Hospital	95 (49.5)	
	Online & DB	10 (5.2)	
	School	57 (29.7)	
	Community or institute	30 (15.6)	
Number of settings	1	107 (55.8)	3.22 ± 8.32 (1~80)
	2~10	64 (33.3)	
	> 10	11 (5.7)	
	Not reported	10 (5.2)	
Number of independent variables	< 3	119 (62.0)	2.36 ± 1.33 (1~10)
	≥ 3	73 (38.0)	
Number of dependent variables	1	112 (58.3)	1.44 ± 0.76 (1~4)
	≥ 2	44 (22.9)	
	Not reported	36 (18.8)	
Type of dependent variables	Health-related behaviors	49 (25.5)	
	Care-related and role outcomes	2 (1.0)	
	Knowledge, attitudes, and competencies	13 (6.8)	
	Physiological and clinical outcomes	30 (15.6)	
	Psychological and psychosocial outcomes	27 (14.1)	
	Quality of life and well-being	14 (7.3)	
	Safety and occupational outcomes	10 (5.2)	
	Sleep and circadian-related outcomes	11 (5.7)	
	Not reported	36 (18.8)	
Statistical methods	Descriptive analysis	9 (4.7)	
Data collection (human)	Regression analysis	74 (38.5)	
	Logistic regression	25 (13.0)	
	Correlation analysis	41 (21.4)	
	Group difference analysis	39 (20.3)	
	SEM	4 (2.1)	

JKBNS = *Journal of Korean Biological Nursing Science*; M = Mean; SD = Standard deviation; SEM = Structural equation modeling; IRB = Institutional review board; DB = Database.

**Table 2.** Item-level Compliance with the STROBE Checklist in Cross-sectional Studies Published in JKBNS (2011~2024) (N = 192)

STROBE checklist		Fully reported studies (n)	Study-level STROBE scores M ± SD (range)
Domains	Sub-items		
Title and abstract	1a. Title	6	20.56 ± 2.27 (13~25)
	1b. Abstract	192	
Introduction	2. Background/rationale	192	
	3. Objectives	192	
Methods	4. Study design	190	
	5. Setting	170	
	6. Eligibility criteria	116	
	7. Clearly define all outcomes	192	
	8. Data sources/measurement	192	
	9. Bias	19	
	10. Study size	96	
	11. Quantitative variables	169	
	12a. Statistical methods: confounding	58	
	12b. Statistical methods: subgroups and interactions	172	
	12c. Statistical methods: missing data	0	
	12d. Statistical methods: sampling strategy	0	
	12e. Statistical methods: sensitivity analyses	0	
Results	13a. Participants: number of individuals	157	
	13b. Participants: reasons for non-participation	153	
	13c. Participants: flow diagram	1	
	14a. Descriptive data: characteristics of participants	179	
	14b. Descriptive data: number of participants	150	
	15. Outcome data	192	
	16a. Main results: unadjusted estimates	185	
	16b. Main results: category boundaries	154	
	16c. Main results: translating estimates	NA	
	17. Other analyses	174	
Discussion	18. Key results	192	
	19. Limitations	92	
	20. Interpretation	192	
	21. Generalizability	98	
Other information	22. Funding	72	
M ± SD		127.32 ± 72.11	
Cohen's kappa		0.99	

STROBE Checklist items include: 1a. title; 1b. abstract; 2. background/rationale; 3. objectives; 4. study design; 5. setting; 6. eligibility criteria; 7. clearly defined outcomes; 8. data sources/measurement; 9. bias; 10. study size; 11. quantitative variables; 12a. statistical methods for confounding; 12b. statistical methods for subgroups and interactions; 12c. statistical methods for missing data; 12d. statistical methods for sampling strategy; 12e. statistical methods for sensitivity analyses; 13a. participants: number of individuals; 13b. participants: reasons for non-participation; 13c. participants: flow diagram; 14a. descriptive data: characteristics of participants; 14b. descriptive data: number of participants; 15. outcome data; 16a. main results: unadjusted estimates; 16b. main results: category boundaries; 16c. main results: translating estimates; 17. other analyses; 18. key results; 19. limitations; 20. interpretation; 21. generalizability; and 22. funding. STROBE = Strengthening the Reporting of Observational Studies in Epidemiology; JKBNS = *Journal of Korean Biological Nursing Science*; M = Mean; SD = Standard deviation; NA = Not applicable.

**Table 3.** Compliance Rates for the 22 STROBE Checklist Items in Cross-sectional Studies Published in JKBNS (2011~2024) (N = 192)

STROBE checklist		Fully reported studies (n)	Study-level STROBE scores M ± SD (range)	STROBE reporting compliance rate (%)
Domains	Sub-items			
Title and abstract	1. Title/abstract	99	16.27 ± 1.72 (11.50~19.87)	74.0 ± 7.8 (52.3~90.3)
Introduction	2. Background/rationale	192		
	3. Objectives	192		
Methods	4. Study design	190		
	5. Setting	170		
	6. Eligibility criteria	116		
	7. Clearly define all outcomes	192		
	8. Data sources/measurement	192		
	9. Bias	19		
	10. Study size	96		
	11. Quantitative variables	169		
	12. Statistical methods	46		
Results	13. Participants flow diagram	104		
	14. Descriptive data	165		
	15. Outcome data	192		
	16. Main results	171		
	17. Other analyses	174		
Discussion	18. Key results	192		
	19. Limitations	92		
	20. Interpretation	192		
	21. Generalizability	98		
Other information	22. Funding	72		
M ± SD		142.05 ± 54.98		

STROBE Checklist items include: 1. Title & abstract; 2. background/rationale; 3. objectives; 4. study design; 5. setting; 6. eligibility criteria; 7. clearly defined outcomes; 8. data sources/measurement; 9. bias; 10. study size; 11. quantitative variables; 12. statistical methods; 13. participants flow diagram; 14. descriptive data; 15. outcome data; 16. main results; 17. other analyses; 18. key results; 19. limitations; 20. interpretation; 21. generalizability; and 22. funding. STROBE = Strengthening the Reporting of Observational Studies in Epidemiology; JKBNS = *Journal of Korean Biological Nursing Science*; M = Mean; SD = Standard deviation.

**Table 4.** Item-level Conceptual Mapping Among the STROBE Checklist, JBI, and NIH Cross-sectional Checklist

STROBE domains	STROBE items	JBI Cross-sectional checklist items	NIH Cross-sectional tool items
Title and abstract	1. Title/abstract		
Introduction	2. Background/rationale		
	3. Objectives		Item 1
Methods	4. Study design		
	5. Setting	Item 2	Item 2, 6, 7
	6. Eligibility criteria	Item 1, 2	Item 2, 4
	7. Clearly define all outcomes	Item 5	Item 11
	8. Data sources/measurement	Item 3, 4, 7	Item 9, 10, 11
	9. Bias	Item 2, 5, 6, 7, 8	Item 3, 11, 12, 14
	10. Study size		Item 5
	11. Quantitative variables	Item 4	Item 8
	12. Statistical methods	Item 6, 8	Item 6, 13, 14
Results	13. Participants flow diagram		Item 3, 15
	14. Descriptive data	Item 6	
	15. Outcome data	Item 8	Item 13
	16. Main results	Item 8	Item 13
	17. Other analyses	Item 8	Item 14
Discussion	18. Key results		
	19. Limitations		
	20. Interpretation		
	21. Generalizability		
Other information	22. Funding		Item 16
Cohen's kappa		0.85	0.90

JBI Cross-Sectional Checklist items include: 1. inclusion criteria defined; 2. subjects and setting described; 3. valid exposure measurement; 4. objective outcome criteria; 5. confounding factors identified; 6. strategies to address confounding; 7. valid outcome measurement; and 8. appropriate statistical analysis. NIH Cross-Sectional Tool items include: 1. research question/objective stated; 2. population defined; 3. participation rate ≥50%; 4. uniform eligibility criteria; 5. sample size justification; 6. exposure measured prior to outcome; 7. sufficient timeframe; 8. exposure level variation; 9. valid exposure measurement; 10. repeated exposure assessment; 11. valid outcome measurement; 12. blinded outcome assessment; 13. loss to follow-up ≤20%; and 14. confounders measured and adjusted. Items were mapped at the STROBE item level based on conceptual alignment; one-to-many or partial mappings were allowed due to differences in scope and purpose between reporting guidelines and methodological quality appraisal tools. STROBE = Strengthening the Reporting of Observational Studies in Epidemiology; JBI Cross-Sectional Checklist = Joanna Briggs Institute Critical Appraisal Checklist for Analytical Cross-Sectional Studies; NIH Cross-sectional Tool = National Institutes of Health Quality Assessment Tool for Observational Cohort and Cross-Sectional Studies.

## Data Availability

All data extracted from published articles are available upon reasonable request.

## Appendix 1.

1. Kim JH, Byeon DH, Kim MJ, Sim SS, Choo HS, Chai GJ, et al. Handwashing and preventive measures for new types of influenza. Journal of Korean Biological Nursing Science. 2011;13(1):16-22.

2. Song MS, Kim SK, Kim NC. A study on the correlation between elderly women’s depression and physical fitness. Journal of Korean Biological Nursing Science. 2011;13(1):37-43.

3. Choi SH, Choi-Kwon S. The metabolic syndrome and associated lifestyle factors among older adults. Journal of Korean Biological Nursing Science. 2011;13(1):53-60.

4. Park MJ, Lee KS, Jeong JS, Kim JH, Choi JA, Shim GS, et al. The prevalence, subtypes and risk factors of irritable bowel syndrome by ROME III among Korean university students. Journal of Korean Biological Nursing Science. 2011;13(1):61-71.

5. Kim NH, Kwon YS, Kim MA, Lee KH, Kwak HW. A study on the nutritional status, symptoms, and information needs in stroke patients with dysphagia. Journal of Korean Biological Nursing Science. 2011;13(1):72-80.

6. Choi JS, An GJ, Park S. Affecting factors on hospital nurses’ practice of disinfection: Focused on alcohol, chlorhexidine gluconate, and povidone iodine. Journal of Korean Biological Nursing Science. 2011;13(3):125-133.

7. Lee H, Choi-Kwon S. Metabolic syndrome and bone mineral density among elderly Korean women. Journal of Korean Biological Nursing Science. 2011;13(2):134-141.

8. Choe SE, Lee SY. Characteristics and risk factors on colorectal polyps by the gender in health screen examinees. Journal of Korean Biological Nursing Science. 2011;13(2):164-173.

9. Kim SS, Shin G, Ki J. Needlestick and sharps injuries of nursing students. Journal of Korean Biological Nursing Science. 2011;13(2):174-178.

10. Park MJ. The cognition, balance, and quality of life in the elderly. Journal of Korean Biological Nursing Science. 2011;13(2):185-192.

11. Yi YJ, Cho KS. Survey on the students’ evaluation and the administrational status of biological nursing science subjects in advanced practice nursing programs. Journal of Korean Biological Nursing Science. 2011;13(3):193-203.

12. Kim MY, Lee EJ. Rest-activity rhythm and sleep pattern in the elderly. Journal of Korean Biological Nursing Science. 2011;13(3):211-219.

13. Kim MS, Kim YH, Kim JS, Yoo YJ, Kim SO, Won DY, et al. Prevalence related characteristics and management status in children with atopic dermatitis in Ulsan. Journal of Korean Biological Nursing Science. 2011;13(3):220-228.

14. Son YJ, Park YR. Relationships between sleep quality, fatigue and depression on health promoting behavior by shift-work patterns in university hospital nurses. Journal of Korean Biological Nursing Science. 2011;13(3):229-237.

15. Song MR, Lee YS. Differences in blood pressure according to body position by age groups. Journal of Korean Biological Nursing Science. 2011;13(3):238-244.

16. Park JY, Kim NH. The relationship between physical activity and insulin resistance in the middle-aged adults. Journal of Korean Biological Nursing Science. 2011;13(3):245-252.

17. Lee KE, Kim NS. Gender differences in factors affecting dietary self-efficacy in fifth and sixth grade elementary school children. Journal of Korean Biological Nursing Science. 2011;13(3):253-261.

18. Cha NH, Kim YK. Relationship between personality and parental rearing attitudes perceived by nursing college students. Journal of Korean Biological Nursing Science. 2011;13(3):283-290.

19. Lee HK, Park YS, Kim HS, Beak SS, Ji HS. One university staff members’ life styles, body mass indices, lipid profiles and plasma glucose levels. Journal of Korean Biological Nursing Science. 2011;13(3):298-306.

20. Kim JS, Hong HS, Na YK. A study of fatigue, depression and sleep disorders in patients with chronic liver disease. Journal of Korean Biological Nursing Science. 2012;14(1):1-7. https://doi.org/10.7586/jkbns.2012.14.1.1

21. Kim KM, Kim OS, Jeon MY. Knowledge and compliance level of the multidrug-resistant organisms of nursing students. Journal of Korean Biological Nursing Science. 2012;14(1):8-15. https://doi.org/10.7586/jkbns.2012.14.1.8

22. Kim SH, Choi-Kwon S. Nutritional status among elderly Korean women and related factors. Journal of Korean Biological Nursing Science. 2012;14(1):16-24. https://doi.org/10.7586/jkbns.2012.14.1.16

23. Son ME, Kim NH. The relationship between cyber leisure activity and autonomic function in high school students. Journal of Korean Biological Nursing Science. 2012;14(1):33-40. https://doi.org/10.7586/jkbns.2012.14.1.33

24. Chu SH, Kang SM, Kim DR, Lee YJ. Perceptions of anticoagulation therapy and medication adherence among patients taking warfarin. Journal of Korean Biological Nursing Science. 2012;14(1):66-75. https://doi.org/10.7586/jkbns.2012.14.1.66

25. Song MS, Yoo YK, Choi CH, Kim NC. The study of self image according to body mass index in middle school students. Journal of Korean Biological Nursing Science. 2012;14(2):77-83. https://doi.org/10.7586/jkbns.2012.14.2.77

26. Kang KJ, Yu SJ, Seo HM, Yu M, Park MS, Jang HC. Factors influencing self management behavior for patients with type 2 diabetes: Comparison of difference between the elderly and adults. Journal of Korean Biological Nursing Science. 2012;14(2):112-121. https://doi.org/10.7586/jkbns.2012.14.2.112

27. Choi SY, Choi-Kwon S. A survey of perceived stress, depression, body mass index and nutrient intakes for soldiers in the army. Journal of Korean Biological Nursing Science. 2012;14(3):147-155. https://doi.org/10.7586/jkbns.2012.14.3.147

28. Kim MH, Park JH, Kim MS. Predictors of drug calculation competence of nursing students. Journal of Korean Biological Nursing Science. 2012;14(3):174-182. https://doi.org/10.7586/jkbns.2012.14.3.174

29. Choi-Kwon S, Choe MA, Kim KS, Yi MS, Suh E, Suh M. Nutritional status, nutrients intakes, and health status of young-old and old-old homebound elderly in Korea. Journal of Korean Biological Nursing Science. 2012;14(3):183-192. https://doi.org/10.7586/jkbns.2012.14.3.183

30. Chu SH, Ko IS, Lee WH, Yoo JS, Kang SM, Jung AY, et al. Factors affecting medication adherence in patients with chronic heart failure. Journal of Korean Biological Nursing Science. 2012;14(3):193-202. https://doi.org/10.7586/jkbns.2012.14.3.193

31. Choe MA, An GJ, Jeong JS. A coorientation analysis of perception on bionursing between clinical nurses and nursing professors. Journal of Korean Biological Nursing Science. 2012;14(3):212-220. https://doi.org/10.7586/jkbns.2012.14.3.212

32. Jang EH, Park YR. Body composition, blood pressure, blood lipids, and glucose according to obesity degree by body fat percentage in female university students. Journal of Korean Biological Nursing Science. 2012;14(4):231-238. https://doi.org/10.7586/jkbns.2012.14.4.231

33. Kim CH, Song JE. The relationships among knowledge, information seeking behavior, and willingness for education about human papillomavirus vaccination in the middle or high school teachers. Journal of Korean Biological Nursing Science. 2012;14(4):239-248. https://doi.org/10.7586/jkbns.2012.14.4.239

34. Byun MS, Kim NH. Energy intake and fatigue in patients receiving chemotherapy. Journal of Korean Biological Nursing Science. 2012;14(4):258-267. https://doi.org/10.7586/jkbns.2012.14.4.258

35. Han MH, Hong HS. The awareness and performance of the forensic nursing role in emergency departments. Journal of Korean Biological Nursing Science. 2012;14(4):291-299. https://doi.org/10.7586/jkbns.2012.14.4.291

36. Sohn HS, Kee MJ, Kang MK, Han YO, Moon KH, Kim DI, et al. The attitude on exercise, physical activity and quality of life in hemodialysis patients. Journal of Korean Biological Nursing Science. 2013;15(1):15-23. https://doi.org/10.7586/jkbns.2013.15.1.15

37. Park S, Choi JS. Comparison of human papillomavirus vaccination status, associated with health belief and knowledge between male and female high school students. Journal of Korean Biological Nursing Science. 2013;15(1):24-32. https://doi.org/10.7586/jkbns.2013.15.1.24

38. Oh DN, Lim KC, Park S. Health effects of exposure to oil-contaminated water using biological markers: Focusing on g village near the area of Daecheon beach. Journal of Korean Biological Nursing Science. 2013;15(2):74-81. https://doi.org/10.7586/jkbns.2013.15.2.74

39. Kim HW, Choi-Kwon S. Quality of life in pre-dialysis patients with chronic kidney disease at glomerular filtration rates. Journal of Korean Biological Nursing Science. 2013;15(2):82-89. https://doi.org/10.7586/jkbns.2013.15.2.82

40. Ryu KH, Son YJ. Impact of cognitive function and self-efficacy on medication adherence of elderly patients with chronic disease. Journal of Korean Biological Nursing Science. 2013;15(3):107-114. https://doi.org/10.7586/jkbns.2013.15.3.107

41. Hong HS, Kim DJ, Kim HJ, Seong HJ, Yoon WJ, Na YK. Nursing student’s awareness of the forensic nurse’s role and needs of forensic nursing education. Journal of Korean Biological Nursing Science. 2013;15(3):115-121. https://doi.org/10.7586/jkbns.2013.15.3.115

42. Kim HS, Kim HR. Comparison of the metabolic syndrome risk factors, physical activity, and diet habits between the fifties and sixties in postmenopausal women. Journal of Korean Biological Nursing Science. 2013;15(3):133-138. https://doi.org/10.7586/jkbns.2013.15.3.133

43. Cho MK, Shin G, Choe MA. A study of clinical nurses’ knowledge, need and clinical performance about pathophysiology. Journal of Korean Biological Nursing Science. 2013;15(3):139-146. https://doi.org/10.7586/jkbns.2013.15.3.139

44. Park HS, Cho GY, Kim DH, Kim SH, Kim MS. The mediating effect of drug calculation confidence in the relationship between interest in medication and drug calculation competency. Journal of Korean Biological Nursing Science. 2013;15(4):155-163. https://doi.org/10.7586/jkbns.2013.15.4.155

45. Park JE, Kim HS, Hong HS. Shortening of nursing record time about real time transmission effect of blood pressure, blood glucose value based on u-healthcare. Journal of Korean Biological Nursing Science. 2013;15(4):164-172. https://doi.org/10.7586/jkbns.2013.15.4.164

46. Kim EH, Kim NH. Comparison of stress level and HPA axis activity of internet game addiction vs. non-addiction in adolescents. Journal of Korean Biological Nursing Science. 2013;15(4):173-183. https://doi.org/10.7586/jkbns.2013.15.4.173

47. Kim EK, Song MR. An analysis of the characteristics and preferences related to a smoking cessation program among smoking college students. Journal of Korean Biological Nursing Science. 2013;15(4):184-192. https://doi.org/10.7586/jkbns.2013.15.4.184

48. Ha JS, Choi-Kwon S. The effect of the immediate postoperative nutritional status in liver transplant recipients in SICU on clinical outcome. Journal of Korean Biological Nursing Science. 2013;15(4):193-201. https://doi.org/10.7586/jkbns.2013.15.4.193

49. Hyun HJ, Kim JH, Ko GY, Park BS, Choi EY, Ahn MH. The relationship among sun-screening agent use, bone health promotion behavior and bone mineral density of female college students. Journal of Korean Biological Nursing Science. 2013;15(4):202-209. https://doi.org/10.7586/jkbns.2013.15.4.202

50. Song MR, Park JS. Analysis of allergy symptoms and quality of life among university students with allergic rhinitis. Journal of Korean Biological Nursing Science. 2013;15(4):264-271. https://doi.org/10.7586/jkbns.2013.15.4.264

51. Lee KS, Choi EO, Jeong JS. Survey of curriculum for 4 subjects (structure and function of human body, clinical microbiology, pathophysiology, & mechanism and effect of drugs) of biological nursing in undergraduate nursing education. Journal of Korean Biological Nursing Science. 2014;16(1):17-25. https://doi.org/10.7586/jkbns.2014.16.1.17

52. Park BH, Lee YM. Risk factors of colon polyps in colonoscopy examinee. Journal of Korean Biological Nursing Science. 2014;16(1):33-40. https://doi.org/10.7586/jkbns.2014.16.1.33

53. Kim CG, Cho MK, Park S. A study on perceived connectivity between pharmacological knowledge and clinical practice, and the need for pharmacology education contents in undergraduate courses among clinical nurses. Journal of Korean Biological Nursing Science. 2014;16(1):41-51. https://doi.org/10.7586/jkbns.2014.16.1.41

54. Choi SH, Kwak CS, Choi-Kwon S. Antioxidant capacity and associated factors during the chronic phase after stroke. Journal of Korean Biological Nursing Science. 2014;16(1):52-59. https://doi.org/10.7586/jkbns.2014.16.1.52

55. Yu SJ, Lee KS, Kim JH, Lim KC, Park JS. Health promotion behavior according to body mass index and self-perception of body weight in female nursing students. Journal of Korean Biological Nursing Science. 2014;16(1):60-68. https://doi.org/10.7586/jkbns.2014.16.1.60

56. Choi H. Undergraduate nursing students’ perceived knowledge and attitudes toward genetics and nursing competencies for genetics. Journal of Korean Biological Nursing Science. 2014;16(2):69-79. https://doi.org/10.7586/jkbns.2014.16.2.69

57. Ha Y, Park H. Relationships between short sleep, obesity, and screen time in high school students. Journal of Korean Biological Nursing Science. 2014;16(2):80-89. https://doi.org/10.7586/jkbns.2014.16.2.80

58. Park JH, Shin G, Kim J. A comparison between the contamination level of uniforms and the nasal staphylococcus aureus colonization before and after the clinical practice of nursing students. Journal of Korean Biological Nursing Science. 2014;16(2):90-97. https://doi.org/10.7586/jkbns.2014.16.2.90

59. Chae YR, Choi DH, Yu SJ. Predictors of poor sleep quality among nursing students. Journal of Korean Biological Nursing Science. 2014;16(2):98-104. https://doi.org/10.7586/jkbns.2014.16.2.98

60. Kim MS, Kim JS, Ha WC. Predictors of drug dosage calculation error risk in newly graduated nurses. Journal of Korean Biological Nursing Science. 2014;16(2):113-122. https://doi.org/10.7586/jkbns.2014.16.2.113

61. Na YK, Hong HS, Suk HJ. Blood biochemical parameters, physical activity, stress and sleep management by body mass index. Journal of Korean Biological Nursing Science. 2014;16(2):133-140. https://doi.org/10.7586/jkbns.2014.16.2.133

62. Park HI, Lee K. Pain management survey of psychiatric unit nurses. Journal of Korean Biological Nursing Science. 2014;16(2):150-156. https://doi.org/10.7586/jkbns.2014.16.2.150

63. Min S, Ha YJ, Moon JY. Effect of forensic education and autopsy attitude of nursing student. Journal of Korean Biological Nursing Science. 2014;16(3):211-218. https://doi.org/10.7586/jkbns.2014.16.3.211

64. Jeon MY, Lee YS, Lim JO, Seol JY, Kim JY, Kim Y. A study on the status of drug use among elderly residents in long-term care facility. Journal of Korean Biological Nursing Science. 2014;16(3):244-250. https://doi.org/10.7586/jkbns.2014.16.3.244

65. Shin G, Cho MK. The knowledge, need, and usage of medical terminology in clinical nursing practice. Journal of Korean Biological Nursing Science. 2014;16(4):276-283. https://doi.org/10.7586/jkbns.2014.16.4.276

66. Kim JS, Hong HS. Construction of model for health-related quality of life of liver cirrhosis patients. Journal of Korean Biological Nursing Science. 2014;16(4):292-299. https://doi.org/10.7586/jkbns.2014.16.4.292

67. Suk HJ, Na YK, Hong HS. Difference in sleep circadian rhythm and sleep quality between normal-weight and obese group. Journal of Korean Biological Nursing Science. 2014;16(4):309-317. https://doi.org/10.7586/jkbns.2014.16.4.309

68. Kim YH, Kim HS. Metabolic syndrome prevalence and lifestyle by age and metabolic syndrome status in women religious. Journal of Korean Biological Nursing Science. 2015;17(1):11-18. https://doi.org/10.7586/jkbns.2015.17.1.11

69. Kim JI, Yang YM, Park JY, Shin HJ. Study on the relationship between skinfold thickness and geriatric depression in older adult woman. Journal of Korean Biological Nursing Science. 2015;17(1):44-49. https://doi.org/10.7586/jkbns.2015.17.1.44

70. Kim KS, Choi-Kwon S, Han K. Structural equation modeling on health status in hospital nurses: Based on the theory of salutogenesis with bio behavioral approach. Journal of Korean Biological Nursing Science. 2015;17(1):50-59. https://doi.org/10.7586/jkbns.2015.17.1.50

71. Lim KC. Correlates of body mass index, perceived health status, and the needs of functional games for the elderly in Korea. Journal of Korean Biological Nursing Science. 2015;17(1):60-70. https://doi.org/10.7586/jkbns.2015.17.1.60

72. Song HJ. Factors associated with lower urinary tract symptoms in patients with type 2 diabetes mellitus. Journal of Korean Biological Nursing Science. 2015;17(1):71-77. https://doi.org/10.7586/jkbns.2015.17.1.71

73. Park JY, Kim N, Kang SH. Analysis of physical activity measured by international physical activity questionnaire and actigraph accelerometer, and participation intention for physical activity of breast cancer survivors. Journal of Korean Biological Nursing Science. 2015;17(2):104-113. https://doi.org/10.7586/jkbns.2015.17.2.104

74. Moon JY, Kim BH. Comparison of the level and side effects of spinal anesthesia with hyperbaric bupivacaine in the supine, lateral, and prone positions. Journal of Korean Biological Nursing Science. 2015;17(2):114-122. https://doi.org/10.7586/jkbns.2015.17.2.114

75. Kim NJ, Hong HS. The correlation analysis of fluid intake, skin hydration and skin pH of college students. Journal of Korean Biological Nursing Science. 2015;17(2):132-139. https://doi.org/10.7586/jkbns.2015.17.2.132

76. Bang MR, Sim SS, Lee DS. Comparison of patient-sitter ward nurses and general ward nurses on work-related musculoskeletal symptoms, occupational stress and nursing work environments. Journal of Korean Biological Nursing Science. 2015;17(2):169-178. https://doi.org/10.7586/jkbns.2015.17.2.169

77. Song SH, Choi-Kwon S, Baek JH, Song KJ, Koh CK. Assessment of nurses' nutritional knowledge and educational needs regarding stroke specific diet regimens. Journal of Korean Biological Nursing Science. 2015;17(3):228-235. https://doi.org/10.7586/jkbns.2015.17.3.228

78. Yun HJ, Ra JS, Jang M. Maternal perception of children's weight, maternal body shape satisfaction, and maternal feeding styles in preschool-aged children. Journal of Korean Biological Nursing Science. 2015;17(3):262-270. https://doi.org/10.7586/jkbns.2015.17.3.262

79. Back J, Jun SE. The relationship of eating habits and trigger foods to symptom severity of irritable bowel syndrome. Journal of Korean Biological Nursing Science. 2015;17(4):297-305. https://doi.org/10.7586/jkbns.2015.17.4.297

80. Kim JH, Kim H. Influences of symptom experience and depression on quality of life in colorectal cancer patients with stoma reversal. Journal of Korean Biological Nursing Science. 2015;17(4):306-314. https://doi.org/10.7586/jkbns.2015.17.4.306

81. Jin BY, Kim S. University students' cough etiquette knowledge and practice to protect droplet infection. Journal of Korean Biological Nursing Science. 2015;17(4):348-355. https://doi.org/10.7586/jkbns.2015.17.4.348

82. Cho YH, Cho MK. The impact of alcohol and caffeine intake on body mass index, alcohol use disorder, and quality of sleep among university freshmen. Journal of Korean Biological Nursing Science. 2015;17(4):363-371. https://doi.org/10.7586/jkbns.2015.17.4.363

83. Kim NJ, Hong HS. Influence of stress, self-efficacy for smoking cessation, smoking temptation and nicotine dependency in male college students who smoke. Journal of Korean Biological Nursing Science. 2016;18(1):1-8. https://doi.org/10.7586/jkbns.2016.18.1.1

84. Lee S, Suh M. Exploring subjective stress, sleep and diurnal variation of salivary cortisol in Korean female adults. Journal of Korean Biological Nursing Science. 2016;18(1):9-16. https://doi.org/10.7586/jkbns.2016.18.1.9

85. Kim EK, Chae YT, Jung YH, Park EH. Assertive behavior in asking smokers not to smoke among patients with vascular diseases. Journal of Korean Biological Nursing Science. 2016;18(1):27-35. https://doi.org/10.7586/jkbns.2016.18.1.27

86. Lim SJ, Lee HJ. The effect of knowledge, attitudes and prevention behaviors for tuberculosis infection in nursing students. Journal of Korean Biological Nursing Science. 2016;18(1):43-50. https://doi.org/10.7586/jkbns.2016.18.1.43

87. Lee SH, Yoo YS. Disease-related knowledge, stress, and quality of life in patients with varicose veins. Journal of Korean Biological Nursing Science. 2016;18(1):60-67. https://doi.org/10.7586/jkbns.2016.18.1.60

88. Kim SO. The relationships between body mass index, nutrition knowledge and the health promotion behavior of nursing students. Journal of Korean Biological Nursing Science. 2016;18(2):87-93. https://doi.org/10.7586/jkbns.2016.18.2.87

89. Seo KH, Eun Y, Jeon MY. Sports injuries and the changes in physical activity, perceived health state and exercise self-efficacy according to the sports injuries of the elderly who participate in physical activities. Journal of Korean Biological Nursing Science. 2016;18(2):102-109. https://doi.org/10.7586/jkbns.2016.18.2.102

90. Yoo SY, Kim OS. Comparison of the incidence rate of influenza-like illness between an influenza-vaccinated group and unvaccinated group. Journal of Korean Biological Nursing Science. 2016;18(2):110-117. https://doi.org/10.7586/jkbns.2016.18.2.110

91. Cho HK, Jeong JS, Moon S, Kim MN. Current immunization status and factors affecting the influenza vaccination in kidney transplant patients. Journal of Korean Biological Nursing Science. 2016;18(2):118-125. https://doi.org/10.7586/jkbns.2016.18.2.118

92. Kim JK, Kim JB, Song MS. A study on physiological index, anxiety and depression by the severity of lower urinary tract symptoms in patients with benign prostatic hyperplasia. Journal of Korean Biological Nursing Science. 2016;18(3):127-134. https://doi.org/10.7586/jkbns.2016.18.3.127

93. Kim JH, Chu SH. Factors associated with obstructive sleep apnea risk in patients with metabolic syndrome. Journal of Korean Biological Nursing Science. 2016;18(3):135-143. https://doi.org/10.7586/jkbns.2016.18.3.135

94. Kim MA. Relationship among pro-environmental attitude, behavior to decrease exposure, knowledge of endocrine disruptors, and obesity-related profiles in nursing students. Journal of Korean Biological Nursing Science. 2016;18(3):160-168. https://doi.org/10.7586/jkbns.2016.18.3.160

95. Kim MY, Choi HJ. Factors influencing sleep patterns during clinical practice weeks among nursing students: Based on Spielman's model. Journal of Korean Biological Nursing Science. 2016;18(4):203-212. https://doi.org/10.7586/jkbns.2016.18.4.203

96. Kim NJ, Hong HS. Influence of knowledge about lung cancer, attitude and preventive health behavior about cancer on nicotine dependency in smoking male college students. Journal of Korean Biological Nursing Science. 2016;18(4):213-220. https://doi.org/10.7586/jkbns.2016.18.4.213

97. Song HY, Nam KA. Sex differences of the relationships between cardiovascular risk markers and psychosocial factors in community-residing adults. Journal of Korean Biological Nursing Science. 2016;18(4):221-230. https://doi.org/10.7586/jkbns.2016.18.4.221

98. Lee HS, Choi M, Oh EG. Factors related to n-terminal pro-b-type natriuretic peptide as a biomarker for heart failure. Journal of Korean Biological Nursing Science. 2016;18(4):247-256. https://doi.org/10.7586/jkbns.2016.18.4.247

99. Park MS, Choi HJ, Kim YJ, Chang HK, Chang SJ, Lee H. The relevance between pathophysiological subject and examination workbook items for national nurse licensure examination in South Korea and the United States. Journal of Korean Biological Nursing Science. 2016;18(4):264-273. https://doi.org/10.7586/jkbns.2016.18.4.264

100. Kim YO, Jeong JS. Adequacy of reprocessing gastrointestinal endoscopes in Korea hospitals. Journal of Korean Biological Nursing Science. 2016;18(4):288-295. https://doi.org/10.7586/jkbns.2016.18.4.288

101. Jeon HO, Kim B, Kim H, Chae MO, Kim MA, Kim A. Factors influencing medication adherence and status of medication use of the elderly with chronic disease taking non-opioid analgesics. Journal of Korean Biological Nursing Science. 2017;19(1):18-29. https://doi.org/10.7586/jkbns.2017.19.1.18

102. Song HY. The airflow obstruction and subjective health status among stable chronic obstructive pulmonary disease patients residing in the community. Journal of Korean Biological Nursing Science. 2017;19(1):38-47. https://doi.org/10.7586/jkbns.2017.19.1.38

103. Ko SJ, Park YS, Kang MJ, Hong HS. Influence of severity of problem drinking, circadian rhythm and sleep quality on sleep disorder in alcohol use disorder patients. Journal of Korean Biological Nursing Science. 2017;19(1):48-54. https://doi.org/10.7586/jkbns.2017.19.1.48

104. Seo JH, Jung EY. Factors influencing nursing students' performance on standard precautions of infection control. Journal of Korean Biological Nursing Science. 2017;19(2):69-75. https://doi.org/10.7586/jkbns.2017.19.2.69

105. Choi DH, Chae YR. The effect of health inequality factors on health level of the rural elderly. Journal of Korean Biological Nursing Science. 2017;19(2):98-106. https://doi.org/10.7586/jkbns.2017.19.2.98

106. Kim SH. Comparison of lipid profile ratios in patients with high-grade brain cancers according to the presence of recurrence during cancer-related therapy. Journal of Korean Biological Nursing Science. 2017;19(2):107-112. https://doi.org/10.7586/jkbns.2017.19.2.107

107. Park AS, Ko E. Influences of rehabilitation motivation, self-efficacy and family support on rehabilitation adherence in stroke patients. Journal of Korean Biological Nursing Science. 2017;19(2):113-122. https://doi.org/10.7586/jkbns.2017.19.2.113

108. Park J, Kim N. Influence of lower urinary tract symptoms, physical activity, and depression on the quality of sleep in elderly women with urinary incontinence. Journal of Korean Biological Nursing Science. 2017;19(3):170-177. https://doi.org/10.7586/jkbns.2017.19.3.170

109. Baek JH, Choi-Kwon S. Sleep patterns, alertness and fatigue of shift nurses according to circadian types. Journal of Korean Biological Nursing Science. 2017;19(3):198-205. https://doi.org/10.7586/jkbns.2017.19.3.198

110. Suh M. An exploratory study on occupational stress and anxiety through salivary cortisol and self-report scale in Korean nurses on shift and regular work. Journal of Korean Biological Nursing Science. 2017;19(3):206-213. https://doi.org/10.7586/jkbns.2017.19.3.206

111. Lee AR, Lim S, Han K. Association of sleep, dietary behaviors and physical activity with quality of life among shiftwork nurses. Journal of Korean Biological Nursing Science. 2017;19(4):252-257. https://doi.org/10.7586/jkbns.2017.19.4.252

112. Kim M, Kim HJ, Shin G. Exercise patterns and factors affecting exercise duration in pregnant women. Journal of Korean Biological Nursing Science. 2017;19(4):258-265. https://doi.org/10.7586/jkbns.2017.19.4.258

113. Lee H, Kim YJ, Chang HK, Chang SJ, Choi H, Park MS. The relevance between biological nursing subjects and registered nurse licensure examination workbook in Republic of Korea and the United States of America. Journal of Korean Biological Nursing Science. 2018;20(1):1-10. https://doi.org/10.7586/jkbns.2018.20.1.1

114. Park E, Oh HJ, Kim SH, Min A. The relationships between particulate matter risk perception, knowledge, and health promoting behaviors among college students. Journal of Korean Biological Nursing Science. 2018;20(1):20-29. https://doi.org/10.7586/jkbns.2018.20.1.20

115. Kang Y, Oh SH, Hong HC. The relationship between sleep quality and stress among nursing students in Korea. Journal of Korean Biological Nursing Science. 2018;20(1):30-37. https://doi.org/10.7586/jkbns.2018.20.1.30

116. Jeong JS, Hwang YH, Kim Y, Ryu JG, Kim MK, Choi SE, et al. Current status of biological nursing science education for clinical nurses in general hospital. Journal of Korean Biological Nursing Science. 2018;20(1):47-53. https://doi.org/10.7586/jkbns.2018.20.1.47

117. Kang K. Influence of awareness of sexual harassment on nursing students' coping behavior during clinical practice. Journal of Korean Biological Nursing Science. 2018;20(2):76-83. https://doi.org/10.7586/jkbns.2018.20.2.76

118. Moon H. Factors affecting HPV vaccination rates of daughters aged 12 years. Journal of Korean Biological Nursing Science. 2018;20(2):114-121. https://doi.org/10.7586/jkbns.2018.20.2.114

119. You SY, Tak YR. Expected family involvement of family of elderly residents in nursing homes. Journal of Korean Biological Nursing Science. 2018;20(3):150-158. https://doi.org/10.7586/jkbns.2018.20.3.150

120. Ryu JG, Choi-Kwon S. Association of sleep disturbance, fatigue, job stress and exposure to blood and body fluid in shift-work nurses. Journal of Korean Biological Nursing Science. 2018;20(3):187-195. https://doi.org/10.7586/jkbns.2018.20.3.187

121. Oh JH, Hwang YH. The effects of empathy on interpersonal relationship through the mediating effect of ego-resilience in nursing students. Journal of Korean Biological Nursing Science. 2018;20(3):196-203. https://doi.org/10.7586/jkbns.2018.20.3.196

122. Kim S, Ryu E. Control effect of illness perception on depression and quality of life in patients with hemodialysis: Using structural equation modeling. Journal of Korean Biological Nursing Science. 2018;20(4):221-227. https://doi.org/10.7586/jkbns.2018.20.4.221

123. Jeong YJ, Kim H. Evaluation of clinical alarms and alarm management in intensive care units. Journal of Korean Biological Nursing Science. 2018;20(4):228-235. https://doi.org/10.7586/jkbns.2018.20.4.228

124. Park JH, Chang SJ, Choi S. Correlation between knowledge, attitude, and compliance of preventive behaviors regarding middle east respiratory syndrome among nursing students. Journal of Korean Biological Nursing Science. 2018;20(4):252-260. https://doi.org/10.7586/jkbns.2018.20.4.252

125. Kim KS, Lee KS. Factors influencing hand dermatitis in nurses. Journal of Korean Biological Nursing Science. 2019;21(1):37-45. https://doi.org/10.7586/jkbns.2019.21.1.37

126. Kim H, Park HR. The effects of organizational culture for infection control and self-efficacy on compliance with standard precautions of emergency room nurses. Journal of Korean Biological Nursing Science. 2019;21(1):46-53. https://doi.org/10.7586/jkbns.2019.21.1.46

127. Jung KH, Chun N. Influence of stress, social support and lifestyle on health-related quality of life in middle aged women. Journal of Korean Biological Nursing Science. 2019;21(1):62-69. https://doi.org/10.7586/jkbns.2019.21.1.62

128. Kim JH, Song Y. The influence of chronotype and self-efficacy on problem drinking in undergraduate students. Journal of Korean Biological Nursing Science. 2019;21(1):70-76. https://doi.org/10.7586/jkbns.2019.21.1.70

129. Ahn JH, Kim HS, Kim HJ. Pain, disability, emotional status and educational needs between acute and chronic low back pain groups. Journal of Korean Biological Nursing Science. 2019;21(1):77-84. https://doi.org/10.7586/jkbns.2019.21.1.77

130. Wi S, Park D, Kim H, Park M, Hong H. The relationship between attitude and coping skills toward secondhand e-cigarette smoking among nonsmoking college students. Journal of Korean Biological Nursing Science. 2019;21(2):114-122. https://doi.org/10.7586/jkbns.2019.21.2.114

131. Sung K. Influencing factors of the metabolic index and cardiovascular risk factors on depressive and non-depressive groups in the vulnerable diabetic elderly women. Journal of Korean Biological Nursing Science. 2019;21(2):123-132. https://doi.org/10.7586/jkbns.2019.21.2.123

132. Ku E, Lee G, Jeon M, Choi J, Lee Y. Microbial contamination of reusable suction container and cost analysis of reusable suction container and disposable suction container. Journal of Korean Biological Nursing Science. 2019;21(2):133-140. https://doi.org/10.7586/jkbns.2019.21.2.133

133. Jeong AY, Choi YH, Choi JH, Kwon SG, Kim HR. Differences in awareness, attitude and knowledge toward muscle health according to general characteristics. Journal of Korean Biological Nursing Science. 2019;21(2):152-159. https://doi.org/10.7586/jkbns.2019.21.2.152

134. Park KS, Yoon HM. Effect of college students' perceived stress, cognitive response to stress, and somatization on heart rate variability. Journal of Korean Biological Nursing Science. 2019;21(3):178-187. https://doi.org/10.7586/jkbns.2019.21.3.178

135. Choi S. Relationships between smartphone usage, sleep patterns and nursing students' learning engagement. Journal of Korean Biological Nursing Science. 2019;21(3):231-238. https://doi.org/10.7586/jkbns.2019.21.3.231

136. Chong HJ. Psychosocial assessment and related factors for kidney transplantation candidates in south Korea: A descriptive correlational study. Journal of Korean Biological Nursing Science. 2019;21(4):249-258. https://doi.org/10.7586/jkbns.2019.21.4.249

137. Park JH, Lee HJ. Clinical nurses' knowledge and educational needs about dizziness. Journal of Korean Biological Nursing Science. 2019;21(4):259-265. https://doi.org/10.7586/jkbns.2019.21.4.259

138. Kil SY, Oh WO, Heo YJ, Suk MH. Mediating effects of sleep quality on the relationship between job stress and stress response of shift-working nurses. Journal of Korean Biological Nursing Science. 2019;21(4):266-274. https://doi.org/10.7586/jkbns.2019.21.4.266

139. Ryu D, Ryu E. Awareness and competency of multi-drug resistant organisms infection control in nursing students with clinical practice. Journal of Korean Biological Nursing Science. 2019;21(4):283-291. https://doi.org/10.7586/jkbns.2019.21.4.283

140. Lee JB, Choi JS. Analysis of prevalence and risk factors for latent tuberculosis infection among healthcare workers. Journal of Korean Biological Nursing Science. 2019;21(4):300-307. https://doi.org/10.7586/jkbns.2019.21.4.300

141. Choi J, Choi-Kwon S. Health promoting behavior and factors in operating room nurses. Journal of Korean Biological Nursing Science. 2019;21(4):308-317. https://doi.org/10.7586/jkbns.2019.21.4.308

142. Baek J, Choi-Kwon S, Park DI, Hong E, Yoon BW. The factors associated with dietary knowledge and educational needs of stroke patients. Journal of Korean Biological Nursing Science. 2020;22(1):61-70. https://doi.org/10.7586/jkbns.2020.22.1.61

143. Chen J, Suh MH. Nutritional intake, body mass index and depression among Chinese college students in an urban area of south Korea. Journal of Korean Biological Nursing Science. 2020;22(2):81-89. https://doi.org/10.7586/jkbns.2020.22.2.81

144. Park BH, Lee KS. Model predicting irritable bowel syndrome severity in university students. Journal of Korean Biological Nursing Science. 2020;22(2):90-101. https://doi.org/10.7586/jkbns.2020.22.2.90

145. Sung G. The effect of oral environment and self-care behavior on oral health-related quality of life in the elderly with diabetes. Journal of Korean Biological Nursing Science. 2020;22(3):192-203. https://doi.org/10.7586/jkbns.2020.22.3.192

146. Park J, Kim S. Role performance and related factors of clinical research nurses in new drug development. Journal of Korean Biological Nursing Science. 2020;22(3):213-221. https://doi.org/10.7586/jkbns.2020.22.3.213

147. Park EH, Choi SE. Support system, stigma and self-care behaviors in patients with pulmonary tuberculosis. Journal of Korean Biological Nursing Science. 2020;22(4):288-296. https://doi.org/10.7586/jkbns.2020.22.4.288

148. Ko E. Factors influencing stroke prevention behaviour in middle-aged adults. Journal of Korean Biological Nursing Science. 2020;22(4):297-307. https://doi.org/10.7586/jkbns.2020.22.4.297

149. Jung BN, Han K, Yoo HY, Chung SJ. The influence of knowledge and sleep hygiene performance on sleep disturbances among shift-work nurses. Journal of Korean Biological Nursing Science. 2020;22(4):308-316. https://doi.org/10.7586/jkbns.2020.22.4.308

150. Suh MH. Differences in heart rate variability depending on sex, level of stress, anxiety, and depression among college students: on the basis of neurovisceral integration model. Journal of Korean Biological Nursing Science. 2021;23(1):22-30. https://doi.org/10.7586/jkbns.2021.23.1.22

151. Jeon HP, An GJ, Lee JH, Lee KM. The influence of infection-related characteristics and patient safety culture on awareness of blood-borne infection prevention in operating room nurses and general ward nurses. Journal of Korean Biological Nursing Science. 2021;23(1):43-54. https://doi.org/10.7586/jkbns.2021.23.1.43

152. Heo YJ, Nam SH, Jeong JS, Kim YH. A comparison of the perception of and adherence to the COVID-19 social distancing behavior guidelines among health care workers, patients, and general public. Journal of Korean Biological Nursing Science. 2021;23(1):55-63. https://doi.org/10.7586/jkbns.2021.23.1.55

153. Hyun JW, Song HJ, Choi JH. Associations of illness symptoms, perception of illness, and coping with quality of life of thyroid cancer patients after thyroidectomy. Journal of Korean Biological Nursing Science. 2021;23(1):83-90. https://doi.org/10.7586/jkbns.2021.23.1.83

154. Lee SJ, Jin X, Lee SJ. Factors influencing COVID-19 preventive behaviors in nursing students: knowledge, risk perception, anxiety, and depression. Journal of Korean Biological Nursing Science. 2021;23(2):110-118. https://doi.org/10.7586/jkbns.2021.23.2.110

155. Seo K, Kim J, An GJ. Current biological nursing curriculum and faculty perceptions on biological nursing subjects in south Korea. Journal of Korean Biological Nursing Science. 2021;23(2):127-137. https://doi.org/10.7586/jkbns.2021.23.2.127

156. Shim H, Sohng KY. Relationship among cognition, sleep patterns, salivary melatonin level and sleep disorder inventory of older adults in nursing homes. Journal of Korean Biological Nursing Science. 2021;23(2):151-158. https://doi.org/10.7586/jkbns.2021.23.2.151

157. Kim NY, Choi HR. The effect of perceived patient- and family-centered care on nurses’ caring behavior in intensive care units. Journal of Korean Biological Nursing Science. 2021;23(3):208-216. https://doi.org/10.7586/jkbns.2021.23.3.208

158. Seo K, Jung MO, Suh M. Agreement of physical activity measured using self-reporting questionnaires with those using actigraph devices, focusing on the correlation with psychological state. Journal of Korean Biological Nursing Science. 2021;23(4):287-297. https://doi.org/10.7586/jkbns.2021.23.4.287

159. Kim M, Moon Y. Factors influencing smoking cessation behavior in patients with ischemic heart disease following coronary angiography. Journal of Korean Biological Nursing Science. 2021;23(4):308-317. https://doi.org/10.7586/jkbns.2021.23.4.308

160. Lee S, Choi JS. Factors influencing COVID-19 AstraZeneca (ChAdOx1) vaccination and side effects among health care workers in an acute general hospital. Journal of Korean Biological Nursing Science. 2021;23(4):318-329. https://doi.org/10.7586/jkbns.2021.23.4.318

161. Lee SH, Park SR, Kim HM, Ko DY, Kang MS, Choi EC, et al. Influencing factors on intention to vaccinate against COVID-19 in college students. Journal of Korean Biological Nursing Science. 2021;23(4):330-338. https://doi.org/10.7586/jkbns.2021.23.4.330

162. Kim JE, Jeong JS, Kim MN, Park ES. Evaluation of environmental contamination and disinfection effects in patient rooms with carbapenem-resistant Enterobacteriaceae using ATP measurements and microbial cultures. Journal of Korean Biological Nursing Science. 2021;23(4):339-346. https://doi.org/10.7586/jkbns.2021.23.4.339

163. Kim M, Kim N. Factors affecting the number of emergency department visits by caregivers of children with fever. Journal of Korean Biological Nursing Science. 2022;24(1):46-57. https://doi.org/10.7586/jkbns.2022.24.1.46

164. Kim MG, Song HJ. The impact of psychological insulin resistance on self-care activities in patients with type 2 diabetes mellitus undergoing insulin therapy. Journal of Korean Biological Nursing Science. 2022;24(1):58-66. https://doi.org/10.7586/jkbns.2022.24.1.58

165. Choi JJ, Chae YR. Role stress, trauma and post-traumatic stress disorder of COVID-19 response task force in public health centers. Journal of Korean Biological Nursing Science. 2022;24(1):67-76. https://doi.org/10.7586/jkbns.2022.24.1.67

166. Park YR, Seo EJ. Correlation among organizational culture, fatigue for infection control, and infection control compliance of COVID-19 among emergency nurses. Journal of Korean Biological Nursing Science. 2022;24(2):104-112. https://doi.org/10.7586/jkbns.2022.24.2.104

167. Jeong E, Lee KS, Yang SK, Cho JH. Influence of health empowerment, spousal support, and post-traumatic growth on health behavior in patients with coronary artery disease. Journal of Korean Biological Nursing Science. 2022;24(2):113-121. https://doi.org/10.7586/jkbns.2022.24.2.113

168. Kim MS, Kang M, Park KH. Factors influencing professional competencies in triage nurses working in emergency departments. Journal of Korean Biological Nursing Science. 2022;24(2):122-130. https://doi.org/10.7586/jkbns.2022.24.2.122

169. Baek MJ, Han K. Association of grit and body composition with fatigue and burnout among shift-work nurses. Journal of Korean Biological Nursing Science. 2022;24(3):141-149. https://doi.org/10.7586/jkbns.2022.24.3.141

170. Lee JL, Jeong Y. Assessing nurses’ educational needs based on knowledge and importance of clinical microbiology. Journal of Korean Biological Nursing Science. 2022;24(3):150-160. https://doi.org/10.7586/jkbns.2022.24.3.150

171. Kim SO, Kim SM. The effects of pressure injury nursing knowledge and pressure injury nursing attitude on pressure injury nursing practices of nurses in geriatric hospitals. Journal of Korean Biological Nursing Science. 2022;24(3):190-199. https://doi.org/10.7586/jkbns.2022.24.3.190

172. Kim JE, Park JH, You MA, Seo EJ. Impact of depression and social support on medication adherence in older adults with multimorbidity. Journal of Korean Biological Nursing Science. 2022;24(3):200-207. https://doi.org/10.7586/jkbns.2022.24.3.200

173. Yuan Y, Jun S. Factors affecting self-management behavior among patients with type 2 diabetes in a border area of southwest China. Journal of Korean Biological Nursing Science. 2022;24(4):219-226. https://doi.org/10.7586/jkbns.2022.24.4.219

174. Kim AY, Kim N. Associations of perceived stress level, serum cortisol level, and telomere length of community-dwelling adults in Korea. Journal of Korean Biological Nursing Science. 2022;24(4):235-242. https://doi.org/10.7586/jkbns.2022.24.4.235

175. Son S, Kim J. The impact of knowledge, risk perception, fear, self-efficacy on COVID-19 preventive behaviors in nursing students. Journal of Korean Biological Nursing Science. 2022;24(4):253-261. https://doi.org/10.7586/jkbns.2022.24.4.253

176. Kim EJ, Kim H. Intensive care unit nurses’ knowledge, attitudes, perceptions of a safe environment, and compliance with the use of personal protective equipment: a descriptive observational study. Journal of Korean Biological Nursing Science. 2023;25(1):63-72. https://doi.org/10.7586/jkbns.23.349

177. Lee J, Kim SO. The influence of e-learning digital literacy on cognitive flexibility and learning flow in nursing students. Journal of Korean Biological Nursing Science. 2023;25(2):87-94. https://doi.org/10.7586/jkbns.23.0001

178. Lee SJ, Lee SJ, Jin X. Factors influencing health-related quality of life for young single-person households: the mediating effect of resilience. Journal of Korean Biological Nursing Science. 2023;25(3):160-171. https://doi.org/10.7586/jkbns.23.0009

179. Song HY, Nam KA. Differences in physical function, self-efficacy, and health-related quality of life by disease severity in community-dwelling patients with chronic obstructive pulmonary disease. Journal of Korean Biological Nursing Science. 2023;25(3):172-182. https://doi.org/10.7586/jkbns.23.0010

180. Kim S, Kim J, Choi MJ, Jeong SH. Evaluation of the applicability of ChatGPT in biological nursing science education. Journal of Korean Biological Nursing Science. 2023;25(3):183-204. https://doi.org/10.7586/jkbns.23.0013

181. Chung G, Kim HJ. Comparison of health literacy and health behaviors between Korean women with and without breast cancer. Journal of Korean Biological Nursing Science. 2023;25(3):205-214. https://doi.org/10.7586/jkbns.23.0012

182. Lee SY, You MA, Ahn JA, Seo EJ. Factors influencing burnout among Korean nurses caring for patients with covid-19: a cross-sectional study. Journal of Korean Biological Nursing Science. 2023;25(4):276-284. https://doi.org/10.7586/jkbns.23.0014

183. Park JY, Choi H. Effects of perceptions of the importance of patient safety management and patient safety competency on patient safety management activities among nurses at unaccredited general hospitals. Journal of Korean Biological Nursing Science. 2024;26(1):60-69. https://doi.org/10.7586/jkbns.24.002

184. Choi HS, Heo ML. The mediating effects of post-pandemic health promotion behaviors in the relationship between anxiety and quality of life in young adults in south Korea: a cross-sectional study. Journal of Korean Biological Nursing Science. 2024;26(2):144-153. https://doi.org/10.7586/jkbns.24.006

185. Beak HJ, Shin G. Factors influencing the level of performance of patient safety nursing activities among hospital nurses. Journal of Korean Biological Nursing Science. 2024;26(2):154-162. https://doi.org/10.7586/jkbns.24.010

186. Shin YY, Lee H. Impacts of death perceptions, terminal care stress, and life satisfaction on attitudes toward end-of-life care among nurses at a tertiary hospital. Journal of Korean Biological Nursing Science. 2024;26(3):218-227. https://doi.org/10.7586/jkbns.24.019

187. Kim KH, Jeong JS. Nurses' vaccination acceptance and related factors in the initial stage of covid-19 vaccination in Korea: a cross-sectional study. Journal of Korean Biological Nursing Science. 2024;26(3):240-249. https://doi.org/10.7586/jkbns.24.013

188. Lee H. Associations of intermediate hyperglycemia with elevated abdominal obesity, high-sensitivity c-reactive protein, and leptin in Korean adults. Journal of Korean Biological Nursing Science. 2024;26(3):250-258. https://doi.org/10.7586/jkbns.24.016

189. Kim SB, Kim YH. Associations of perceptions of patient safety culture, job crafting, and perceptions of patient rounding with patient safety management activities among tertiary hospital nurses. Journal of Korean Biological Nursing Science. 2024;26(3):259-269. https://doi.org/10.7586/jkbns.24.020

190. Park JE, Kim HJ. Factors influencing attitudes toward end-of-life care among Korean emergency room nurses: A descriptive survey study. Journal of Korean Biological Nursing Science. 2024;26(4):373-381. https://doi.org/10.7586/jkbns.24.032

191. Kim JE, Lee SJ. Factors influencing the care burden among family caregivers using dementia care centers for older adults with dementia in Korea: a cross-sectional descriptive study. Journal of Korean Biological Nursing Science. 2024;26(4):382-392. https://doi.org/10.7586/jkbns.24.025

192. Kim H, Choi S. The effects of nurses' spiritual well-being and death awareness on end-of-life nursing attitudes in Korea: a cross-sectional study. Journal of Korean Biological Nursing Science. 2024;26(4):393-402. https://doi.org/10.7586/jkbns.24.023
